# Individualised human phenotype ontology gene panels improve clinical whole exome and genome sequencing analytical efficacy in a cohort of developmental and epileptic encephalopathies

**DOI:** 10.1002/mgg3.2167

**Published:** 2023-03-26

**Authors:** Olivia J. Henry, Tommy Stödberg, Sofia Båtelson, Chiara Rasi, Henrik Stranneheim, Anna Wedell

**Affiliations:** ^1^ Department of Molecular Medicine and Surgery Karolinska Institutet Stockholm Sweden; ^2^ Department of Women's and Children's Health Karolinska Institutet Stockholm Sweden; ^3^ Department of Pediatric Neurology Karolinska University Hospital Stockholm Sweden; ^4^ Science for Life Laboratory, Department of Microbiology, Tumour and Cell Biology Karolinska Institutet Stockholm Sweden; ^5^ Centre for Inherited Metabolic Diseases Karolinska University Hospital Stockholm Sweden

**Keywords:** deep phenotyping, epilepsy, human phenotype ontology, precision medicine, whole exome sequencing, whole genome sequencing

## Abstract

**Background:**

The majority of genetic epilepsies remain unsolved in terms of specific genotype. Phenotype‐based genomic analyses have shown potential to strengthen genomic analysis in various ways, including improving analytical efficacy.

**Methods:**

We have tested a standardised phenotyping method termed ‘Phenomodels’ for integrating deep‐phenotyping information with our in‐house developed clinical whole exome/genome sequencing analytical pipeline. Phenomodels includes a user‐friendly epilepsy phenotyping template and an objective measure for selecting which template terms to include in individualised Human Phenotype Ontology (HPO) gene panels. In a pilot study of 38 previously solved cases of developmental and epileptic encephalopathies, we compared the sensitivity and specificity of the individualised HPO gene panels with the clinical epilepsy gene panel.

**Results:**

The Phenomodels template showed high sensitivity for capturing relevant phenotypic information, where 37/38 individuals' HPO gene panels included the causative gene. The HPO gene panels also had far fewer variants to assess than the epilepsy gene panel.

**Conclusion:**

We have demonstrated a viable approach for incorporating standardised phenotype information into clinical genomic analyses, which may enable more efficient analysis.

## Graphical abstract

We demonstrate the utility of a standardised phenotype‐based genomic analysis method to improve analytical efficacy of whole exome and genome sequencing in an epilepsy cohort. The generated individualised human phenotype ontology gene panels had superior specificity and comparable sensitivity to the epilepsy disease gene panel.

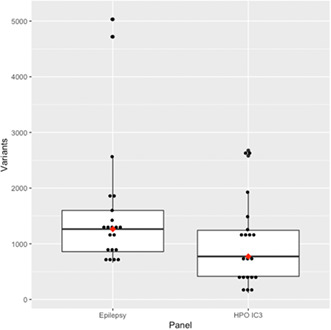

## 1 BACKGROUND

Epilepsy is a heterogeneous disorder in terms of clinical presentation, prognosis and aetiology, particularly when considering genetic aetiologies. The updated International League Against Epilepsy (ILAE) classifications explicitly state for aetiology to be considered at all stages of classification including seizure type, epilepsy type and epilepsy syndrome (Fisher et al., 2017; Scheffer et al., 2017), highlighting a shift towards precision medicine based care.

In‐depth phenotypic data greatly informs the interpretation of genomic data and facilitates gene discovery. Indeed, the first epilepsy gene, *CHRNA4*, was identified following thorough phenotyping of a large family with autosomal dominant nocturnal frontal lobe epilepsy (Steinlein et al., 1995). Phenotype–genotype correlations have helped uncover the now many established genetic epilepsy syndromes, although there are many instances where these associations are not straight forward (Perucca et al., 2020; Wang et al., 2017). Individuals presenting with severe forms of epilepsy called developmental and epileptic encephalopathies (DEEs) can be particularly challenging to characterise due to the heterogeneity of both clinical manifestations and molecular pathogenesis (Maver et al., 2016; Perucca et al., 2020). Detailed phenotyping has been an essential tool in identifying disease genes in severe epilepsies. One of the earlier large whole exome sequencing (WES) epilepsy studies systematically phenotyped 264 individuals with DEEs, leading to the discovery of three now established DEE genes (Epi4K Consortium; Epilepsy Phenome/Genome Project, et al., 2013). Further studies employing WES in DEEs have revealed yields from 20% to 60% (Allen et al., 2016; Helbig et al., 2016; Jiang et al., 2021; Musante et al., 2022; Palmer et al., 2018), highlighting the significant proportion of DEE aetiologies which remain to be uncovered.

Documenting phenotypic information in a more systematic and standardised manner would enable this information to be leveraged for improved genomic analysis. The Human Phenotype Ontology (HPO) is a resource which can be utilised for standardised documentation of clinical phenotypic information. The HPO is comprised of a large, standardised vocabulary which describes phenotypic traits encountered in human disease (Bastarache et al., 2018; Kohler et al., 2017). At the time of writing the current HPO from the 11 June 2022 release contains 16,945 terms with a total of 4526 annotated genes which are mined from OMIM, DECIPHER, Orphanet21 and the medical literature (Bastarache et al., 2018; Kohler et al., 2017). These traits form an interoperable disease network which is organised in a directed‐acyclic‐graph: a hierarchical‐like structure which enables direct relationships between phenotypic traits of varying levels of detail to be formed.

Human Phenotype Ontology based analyses have the potential to improve gene discovery and precision medicine in DEEs. Helbig et al. (2019) identified a novel DEE gene through analysing the HPO signatures of the participants. HPO term specificity was calculated through its information content (IC) as defined as the negative logarithm of the terms' usage within the population of 314 DEEs in the study. An IC‐based phenotypic similarity score enabled a statistical comparison of the relatedness of individuals' phenotypes, identifying two individuals with statistically similar phenotypes who also harboured an identical variant in the novel DEE gene *AP2M1* (Helbig et al., 2019). HPO terms have also been exploited to harmonise phenotypic data from large epilepsy WES studies and explore gene‐specific phenotypic signatures. The same phenotype similarity technique employed in the Helbig et al. (2019) study was applied to reveal subsets of individuals with significant phenotypic similarity and shared genetic aetiologies, providing statistical support for genotype–phenotype associations in these cohorts (Galer et al., 2020). HPO term similarity analyses may also facilitate delineation of the phenotypic spectrum of disease genes. HPO term similarity analyses in the DEE gene *SCN2A* enabled quantifiable associations between phenotypes and distinct variant localisations, leading to novel genotype–phenotype correlations (Crawford et al., 2021). For an in‐depth review of HPO‐based computational analyses in neurodevelopmental disorders, see Lewis‐Smith et al. (2022).

The best way to incorporate HPO‐phenotyping into a research or clinical workflow is unclear.

We have performed a pilot study in a cohort of DEEs using a systematic and standardised phenotyping framework, termed Phenomodels, in the analysis of WES and whole genome sequencing (WGS) data. Phenomodels is a method which utilises an HPO‐based epilepsy phenotyping template for systematic documentation of clinical data, as well as an objective measure for generating individualised HPO‐gene panels. We aimed to explore the efficacy of Phenomodels, in combination with our bioinformatics Mutation Identification Pipeline (MIP), by assessing the ability of the epilepsy phenotyping template to capture a sufficient depth of phenotypic information and the specificity of resulting individualised HPO‐gene panels. Further, we provide a brief summary of the literature and outline a potential framework for incorporating systematic phenotype based genomic analysis into the clinical environment, as illustrated by this pilot study.

## 2 METHODS

We performed a retrospective pilot study on DEE patients previously solved through clinical WES/WGS to test the utility of incorporating a standardised, deep phenotyping approach for the analysis of WES/WGS data.

### 2.1 Study cohort

The cohort includes 38 patients with DEEs from the Stockholm region who were referred for clinical diagnostic testing at the Karolinska University Hospital between the years of 2013–2020. Patients received trio (proband and both parents) WES or WGS where parents were available, and were otherwise analysed as singletons. The inclusion criteria included individuals with DEEs (as defined by the updated ILAE classification) (Scheffer et al., 2017) harbouring de novo single nucleotide variants (SNVs) or insertions and deletions (INDELS), classified as pathogenic or likely pathogenic according to the ACMG guidelines, in established DEE genes identified through the precompiled and regularly updated clinical epilepsy gene panel. Legal guardians provided consent for the genomic investigations.

### 2.2 Genomic medicine Centre Karolinska for rare diseases (GMCK‐RD)

Our centre, GMCK‐RD, is the main clinical centre in Sweden for investigating patients with rare disease using whole genome sequencing (Stranneheim et al., 2021). GMCK‐RD, interconnecting the Clinical Genomics facility at Science for Life Laboratory with three different clinics at the Karolinska University Hospital (Clinical Genetics, Centre for Inherited Metabolic Diseases (CMMS) and Clinical Immunology), has developed MIP for the analysis of clinical WES and WGS data (Stranneheim et al., 2014; Stranneheim et al., 2021). MIP is regularly updated to be in line with current best practices and to incorporate innovations which improve analytical efficacy. GMCK‐RD comprises multidisciplinary specialised teams who are responsible for the interpretation of the sequencing output. MIP output, which consists of VCF files containing the variants called for the analysed individuals, can be filtered by genes associated to HPO terms using the in house developed graphical user interface, Scout (Stranneheim et al., 2021). Scout thus provides a feasible platform for incorporating phenotypic HPO data into the clinical genomic analytical workflow.

### 2.3 Mutation identification pipeline (MIP)

WES and WGS was performed in accordance with previously published methods (Stranneheim et al., 2014; Stranneheim et al., 2021) and the output was processed with MIP. MIP has been utilised since 2010 expanding from initial optimisation for the detection of SNVs and INDELS to now include analyses of structural variants, uniparental disomy, repeat expansions, and *SMN1* and *SMN2* copy number analysis (Stranneheim et al., 2021). MIP uses custom developed as well as pre‐existing open‐source tools for variant calling, annotation, frequency comparisons, familial relationships, as previously described (Stranneheim et al., 2014; Stranneheim et al., 2021). Each variant receives a prioritisation rank score based on a model of weighted sums which incorporate Mendelian inheritance pattern, conservation, rarity and predicted protein impact as the final step in the bioinformatic analysis. MIP output is uploaded to the browser‐based decision support software platform Scout, where a rank score is used to distinguish variants with a higher predicted likelihood of causing disease. CMMS at GMCK‐RD performs the clinical epilepsy patient analyses, including curating and regularly updating the clinical epilepsy gene panel (484 genes at last update) through identifying genes with a clear link to epilepsy by publication in a peer review journal or through GMCK‐RD research. WES/WGS variants appearing in Scout can have multiple filters applied, including various clinical and individualised HPO panels, enabling flexible review of the WES/WGS results. For epilepsy patients, trios are analysed when both parental samples are available. Variants are assessed by clinicians and specialists in Scout in a stepwise fashion, with consideration of deep phenotyping information including clinical semiology and investigations (neurophysiology tests, imaging studies, biochemical testing, neuropsychology evaluation, histopathology, etc.) at each step. Variants are initially filtered using the clinical epilepsy gene panel, and when deemed appropriate also the inborn errors of metabolism (IEM) gene panel (currently consisting of 1012 genes). If this step fails to identify a promising variant(s), reanalysis may be performed through manually selecting terms for HPO‐term based filtering. If one or more causative variant(s) are still not identified and there is a high suspicion of rare genetic disease, research‐setting analysis of the whole genome can be offered to the patients/families.

### 2.4 Phenomodels: standardised epilepsy phenotyping template

**FIGURE 1:**
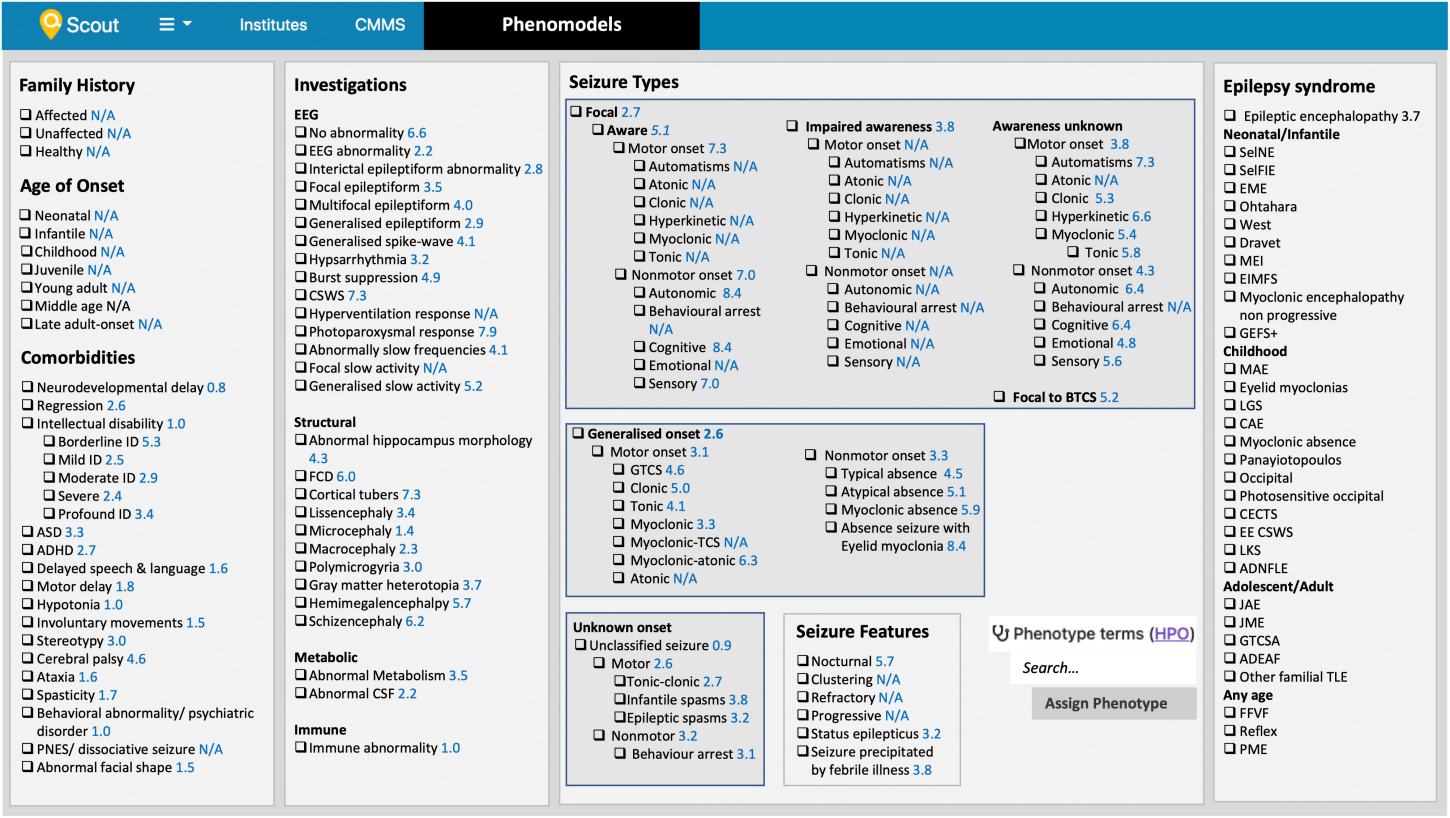
The Phenomodels standardised epilepsy phenotyping template. Blue indicates the information content of the term. ADEAF, Autosomal dominant epilepsy with auditory features; ADNFLE, Autosomal dominant nocturnal frontal lobe epilepsy; CAE, childhood absence epilepsy; CECTS, childhood epilepsy with centrotemporal spikes; EE CSWS, Epileptic encephalopathy with continuous spike‐and‐wave during sleep; EIMFS, epilepsy of infancy with migrating focal seizures; EME, early myoclonic encephalopathy; FBTC, focal to bilateral tonic clonic seizure; FCD, focal cortical dysplasia; FFVF, Familial focal epilepsy with variable foci; GEFS+, genetic epilepsy with febrile seizures plus; GTCSA, Generalised tonic clonic seizures alone; JAE, Juvenile absence epilepsy; JME, Juvenile myoclonic epilepsy; LGS, Lennox Gastaut syndrome; LKS, Landau Kleffner Syndrome; MAE, epilepsy with myoclonic atonic seizures; MEI, myoclonic epilepsy in infancy; Other familial TLE, Other familial temporal lobe epilepsies; PME, Progressive myoclonic epilepsies; SelFIE, self‐limited familial infantile epilepsy; SelNE, self‐limited neonatal epilepsy.

We created a ‘Phenomodels’ epilepsy phenotyping template to incorporate standardised and structured phenotypic information into genomic analysis. The template reflects the updated International League Against Epilepsy (ILAE) classifications (Fisher et al., 2017; Scheffer et al., 2017) and is composed of seven sub‐sections: Family history, Age of Onset, Comorbidities, Investigations, Seizure Types, Seizure Features and Epilepsy Syndrome (Figure 1). The checkbox template format and sub‐section organisation was designed to encourage efficient documentation of phenotypic data. Seizure types include all those outlined in the expanded version of the ILAE classifications (Fisher et al., 2017) and are organised in a hierarchical layout to enable classification according to the depth of information available. Other terms were chosen either for (1) being characteristic traits of specific epilepsy syndromes and/ or (2) their usage in large epilepsy cohorts combining phenotype based‐analyses with WES data (Galer et al., 2020; Helbig et al., 2019), Table S1. Information content (IC) of terms, calculated as described below, was also considered, where terms with a higher IC are preferred when choosing between multiple terms containing overlapping information. The Phenomodels template was used to document the current cohort's medical records from the time of presentation at Karolinska University Hospital up to the molecular diagnosis. The records were reviewed by paediatrician SB and researcher OH and transcribed to the equivalent HPO term on the Phenomodels template. Any cases with unclear presentations were also reviewed by senior paediatric neurologist TS. Prominent individual phenotypic traits which were not included in the Phenomodels template were manually assigned to the case's phenotypic signature using Scout's HPO term search function.

### 2.5 Phenomodels calculations: information content and rank ratio

Information content was used as a guide for selecting informative HPO terms to include in the individualised HPO gene panels. More general terms with a high number of gene associations return a low IC, while HPO terms associated with few genes return a high IC, representing a more specific HPO term. IC was calculated according to Resnik (Resnik, 1995), as follows: $\mathrm{IC}=\ln\left(\frac{N}{n}\right)$, where *N* = total number of genes annotated to the HPO term Phenotypic abnormality (HP:0000118) and *n* = number of genes annotated to the selected HPO term using the 8 August 2021 HPO release (https://github.com/obophenotype/human‐phenotype‐ontology/releases/tag/v2021‐08‐02).

Rank ratio was used to compare the ranking efficacy of panels. Rank ratio is preferable over raw rank as it allows unbiased comparisons between filtering tools which may not return the same number of candidate variants, even when fed the same inputs. Larger rank ratios indicate lower probe variant (i.e. the disease‐causing variant) ranking per total number of variants and poorer performance.

Rank ratio is calculated according to the following equation:

$\mathrm{RR}=\frac{p}{N}\times 100$, where *p* = probe variant rank and *N* = total number of candidate variants (Bornigen et al., 2012).

### 2.6 Phenomodels: creating individualised HPO panels

Terms documented in the Phenomodels template were uploaded to each individuals' case page in Scout. Individualised HPO gene panels were generated through selecting from the HPO terms allocated at the case level which met the following IC thresholds: ≥3, ≥3.25, ≥3.5, ≥3.75 and ≥4. The resulting individualised HPO gene panels were compared across each IC threshold. The 8 August 2021 release of HPO was used to generate HPO gene panels, which were compared with clinical Epilepsy and IEM panel versions from 2017 to 2021, with total genes in the clinical Epilepsy panels ranging from 222 to 427 and clinical IEM panels containing from 723 to 972 genes.

## 3 RESULTS

### 3.1 Overall cohort results

The WGS cohort included 21 DEEs and family members (20 trios, 1 singleton). WGS probands had a median of 9879 total variants (average: 12,022, range: 5323–36,503), a median unfiltered MIP rank of the causative variant of 3 (average: 16.5, range: 1–99) and a median of 12 allocated HPO terms (average: 12, range: 3–20). The WES cohort included 17 DEE patients and their families (14 trios, 3 singletons). WES probands had a median of 820 total variants (average: 830, range: 560–1564), a median unfiltered MIP rank of 4 (average: 12, range: 1–79) and a median of 15 allocated HPO terms (average: 14, range: 6–22). All patients (38/38) had clinical epilepsy panels analysed and 21/38 also had clinical IEM panels analysed.

### 3.2 Whole genome sequencing cohort individualised HPO panels vs. clinical epilepsy panel performance

The IC3 panel captured the causative variant in 95.2% of cases (20/21 patients). All patients' variants were captured in the clinical epilepsy panel (21/21). Increasing the IC threshold decreased the sensitivity for capturing the causative gene (IC3.25 = 90.5%, IC3.5 = 81.0%, IC3.75 = 61.9%, IC4 = 52.4%). IC thresholds lower than 3 still resulted in the exclusion of the single case, thus IC ≥ 3 was deemed an acceptable threshold due to having high specificity and acceptable sensitivity. The median number of variants in the IC3 panel was 773 variants (116–2675), while the epilepsy panel had a median of 1264 variants (range: 661–5031, Figure 2). The median number of HPO terms used in the IC3 panel was 5 (range: 1–13). The HPO IC3 panel had an average rank of the causative variant of 1.60 (median: 1, range: 1–7) and median rank ratio of 0.011 (range: 0.003–0.063), while the clinical epilepsy panel had an average rank of 2.05 (median: 1, range: 1–7) and median rank ratio of 0.014 (range: 0.005–0.212) (Figure 3).

**FIGURE 2:**
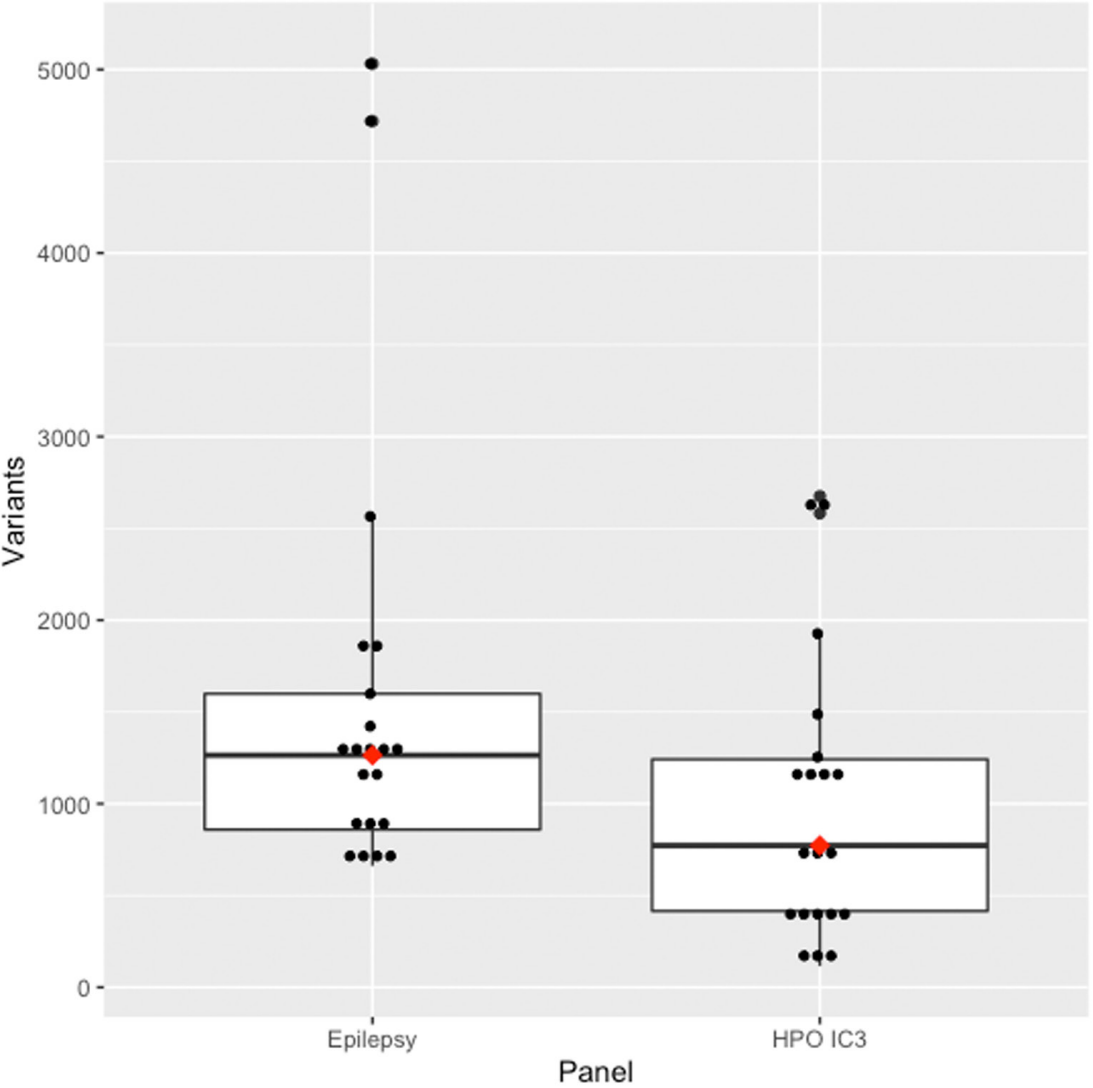
Total whole genome sequencing variants in the clinical epilepsy gene panel and the individualised HPO IC ≥ 3 gene panels. The red dots indicate the median value. The box contains the interquartile range. Values outside the whiskers are outliers.

**FIGURE 3:**
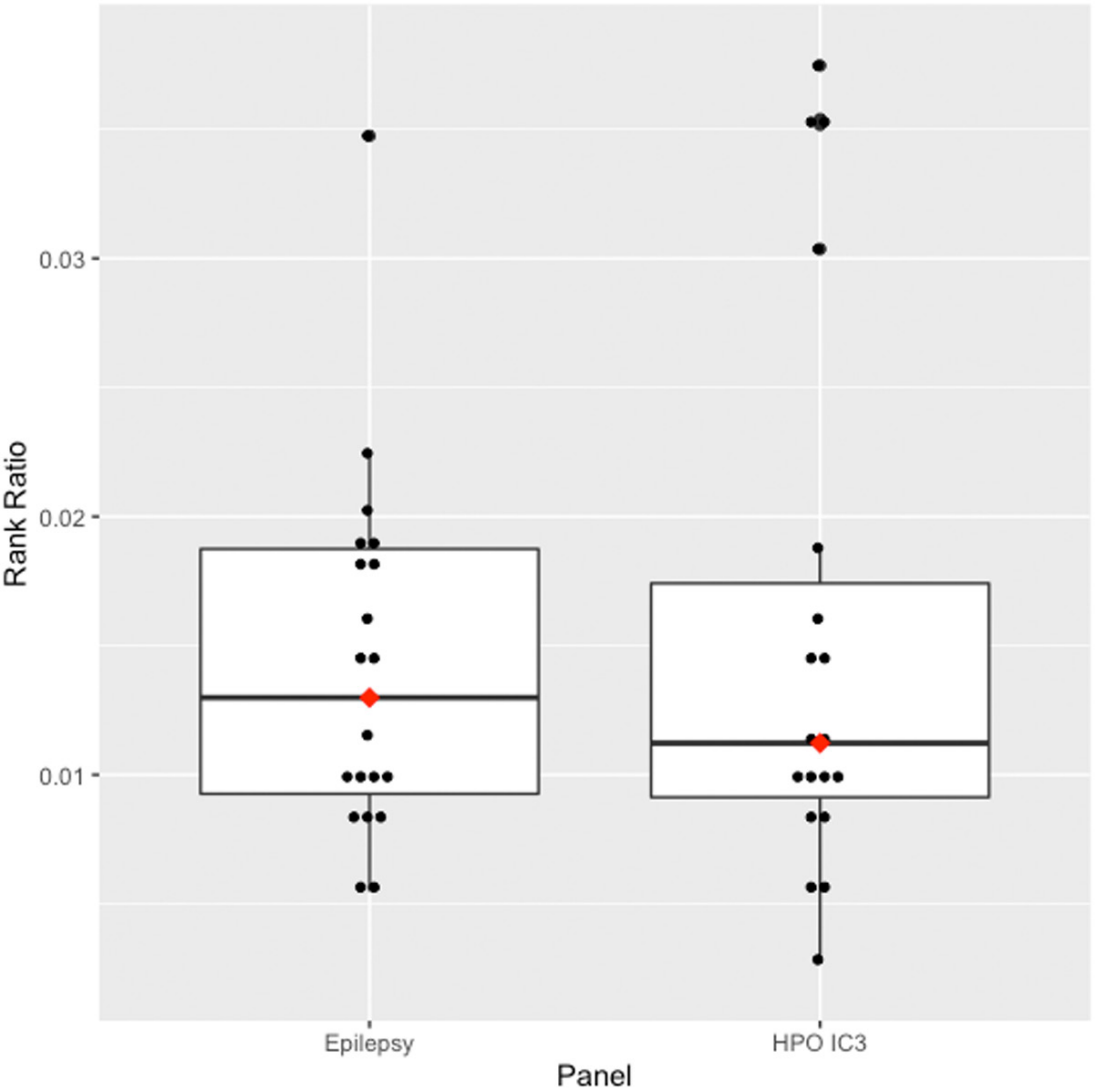
Whole genome sequencing rank ratio in the clinical epilepsy gene panel and the individualised HPO IC ≥ 3 gene panels. Red dots indicate median value. The box contains the interquartile range. Values outside the whiskers are outliers. Extreme outliers 0.212 from the epilepsy gene panel and 0.063 from HPO ≥ 3 gene panel have been removed for visualisation purposes.

For individuals who had clinical epilepsy and IEM panels analysed (10/21), the combined median variants rose to 2166 (range: 1227–8155) (Figure S1), the average rank rose to 3.10 (range: 1–9) and the median rank ratio was 0.018 (range: 0.009–0.071) (Figure S2).

### 3.3 Whole exome sequencing cohort individualised HPO panel vs. clinical epilepsy panel performance

The HPO IC panel was compared across the same 4 thresholds as described for WGS. All cases (17/17) were included in the IC3, 3.25, 3.5 and 3.75 panels, where 1 case was excluded in the IC4 panel. IC3.75 is utilised for comparisons due to its high specificity and sensitivity. All patients' causative variant was captured in the clinical epilepsy panel (17/17).

**FIGURE 4:**
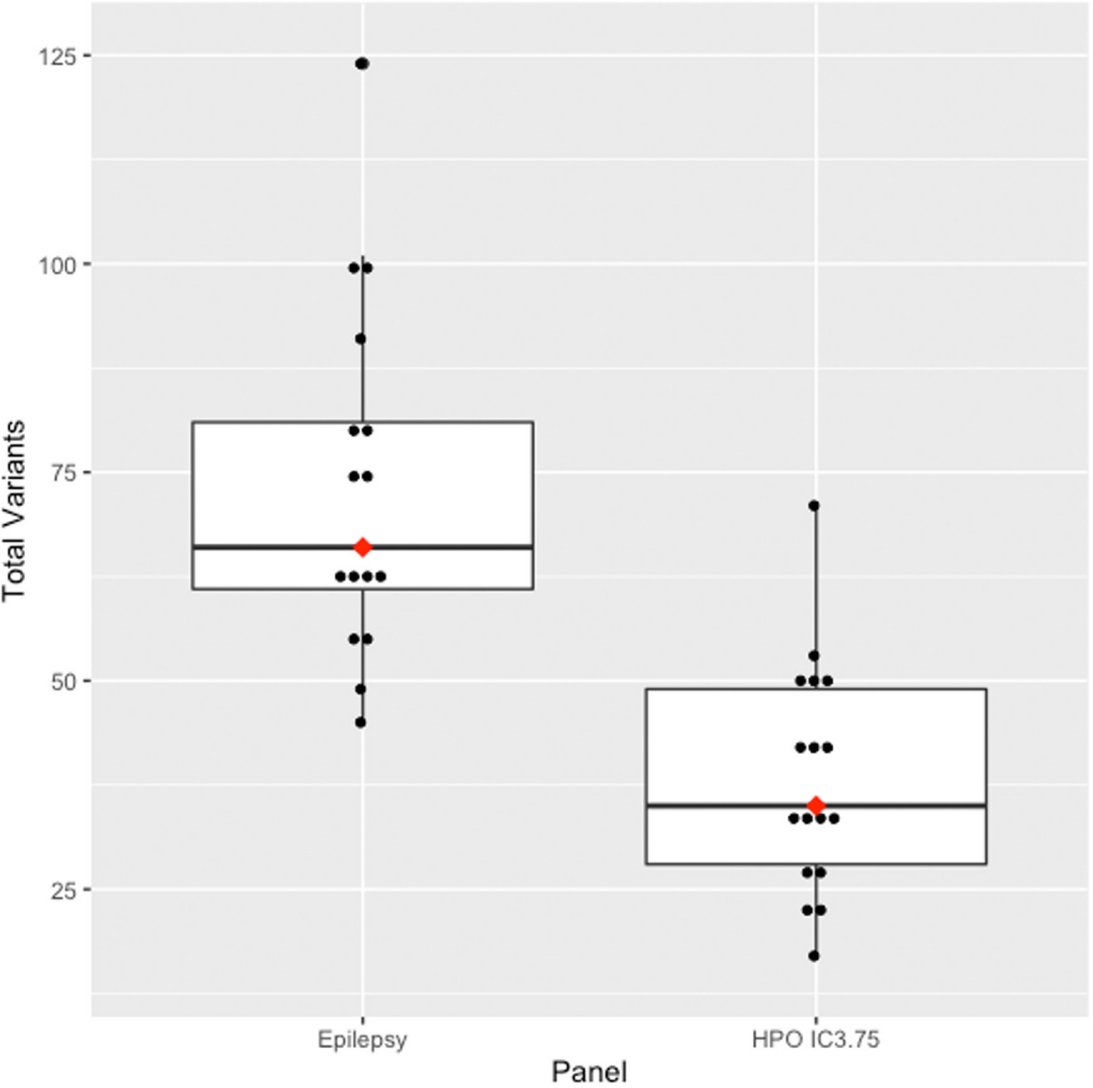
Total whole exome sequencing variants in the clinical epilepsy gene panel and the individualised HPO IC ≥ 3.75 gene panels. The red dots indicate the median value. The box contains the interquartile range. Values outside the whiskers are outliers.

**FIGURE 5:**
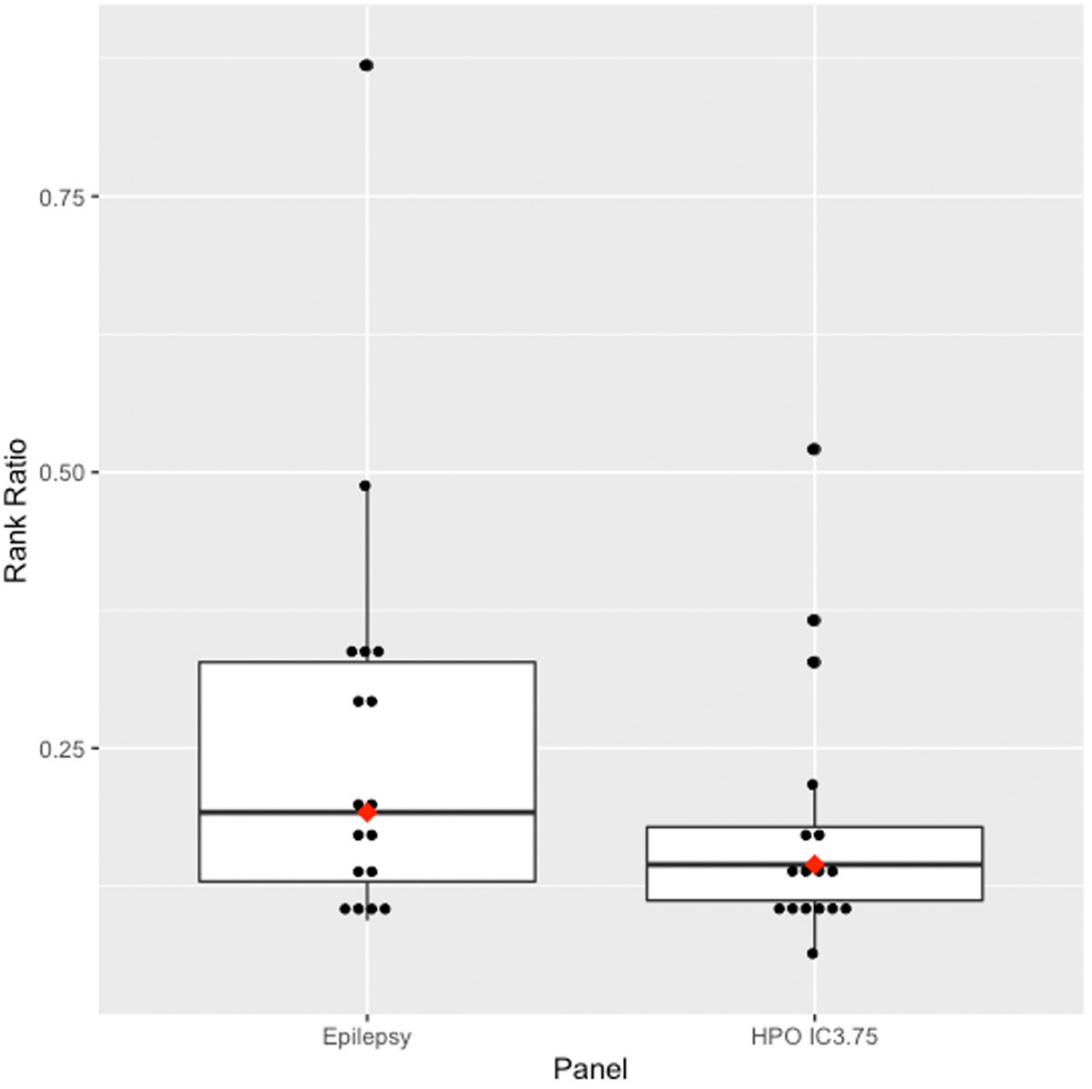
Whole exome sequencing rank ratio in the clinical epilepsy gene panel and the individualised HPO IC ≥ 3.75 gene panels. The red dots indicate the median value. The box contains the interquartile range. Values outside the whiskers are outliers.

The HPO IC3.75 panel included a median of 35 variants (range: 17–71) while the epilepsy panel had a median of 66 variants (range: 45–124) (Figure 4). The HPO IC3.75 panel average rank was 1.35 (median: 1, range: 1–3) and median rank ratio was 0.145 (range: 0.064–0.521), while the epilepsy panel average rank and median rank ratio of the causative variant were 2.12 (median: 2, range: 1–8) and 0.192 (range: 0.094–0.869) (Figure 5). The median number of HPO terms in the IC3.75 panel was 6 (range: 2–11).

For individuals who had clinical epilepsy and IEM panels analysed (11/17) the combined median variants rose to 185 (range: 135–315) (Figure S3), the average rank of the causative variants rose to 3.73 (range: 1–16) and the median rank ratio was 0.256 (range: 0.094–1.737) (Figure S4).

## 4 DISCUSSION

Our clinical pilot study in DEEs shows that applying a systematic, standardised phenotyping approach enables curation of individualised HPO gene panels with comparable sensitivity for capturing the causative gene, yet greater specificity (i.e. fewer unrelated variants), than precompiled clinical epilepsy panels. Further, the rank ratio of the causative variant was lower in the individualised HPO panels than the epilepsy panel, indicating that individualised HPO panels may enable better prioritisation of the causative variant closer to the top of the list respective to the total number of variants.

Despite considerable advances in technologies available for investigating disease semiology, the clinical documentation of phenotypic traits remains comparatively stagnant: stored in an unstandardised format, using free‐text and subjective language. The variability of this information means it must first be translated to standardised terms in order to be used for phenotype‐based analysis of genomic data.

The HPO enables standardised documentation of phenotypic traits, including in depth annotations of epilepsy semiology (Lewis‐Smith et al., 2021), for incorporation with genomic analyses. Prior studies have also found HPO panels to perform better than disease gene panels, with higher inclusion of the causative gene (Kernohan et al., 2018; Maver et al., 2016) as well as a reduced number of variants for assessment (Kernohan et al., 2018). Two other studies found that WGS analysis using individualised HPO‐gene panels led to a diagnosis in 17/38 (45%) (Sanford et al., 2020) and 10/24 (42%) (Mestek‐Boukhibar et al., 2018) of the paediatric intensive care cases, although the yield of disease gene panels was not compared in these cohorts. Algorithms incorporating phenotypic information have also demonstrated improved performance in WES analytical pipelines (Cipriani et al., 2020; Smedley & Robinson, 2015).

The format for incorporating this tool into the clinical workflow is unclear. The number of HPO terms in the panels has been proposed as a measure for obtaining sufficient sensitivity in HPO gene panels. Genomic analysis of patients with varying genetic conditions have found 4–5 HPO terms to deliver the desired phenotypic sensitivity (Galer et al., 2020; Kernohan et al., 2018; Maver et al., 2016; Trakadis et al., 2014; Zemojtel et al., 2014), although these studies provide only vague directives such as “the most relevant/specific, etc., term” for selecting HPO terms to use for panel generation. Contrastingly, one study found noise terms (terms unrelated to the causative gene) reduced the performance of the HPO‐based gene prioritisation algorithm (Masino et al., 2014) and that consistent high performance was related to the specificity of HPO terms used as quantified by IC, rather than the number of HPO terms (Masino et al., 2014). This highlights an additional application of IC to objectively distinguish between HPO terms, where terms with a higher IC are more ‘specific’ and potentially informative terms.

We demonstrate that IC thresholds may be applied to select the most specific terms to include in an individualised HPO gene panel, enabling the investigator to focus on more rare, distinguishing phenotype–genotype associations and avoid excessively long gene panels. Our DEEs had a median of 12 HPO terms each, however upon applying optimal IC thresholds which included nearly all causative variants while greatly reducing the number of variants to assess, the WGS and WES HPO panels contained medians of 5 and 6 HPO terms per panel, respectively, consistent with previous reports (Galer et al., 2020; Kernohan et al., 2018; Maver et al., 2016; Trakadis et al., 2014; Zemojtel et al., 2014). This suggests that IC may provide a more objective guide to implementing previous recommendations to choose the “most specific/ appropriate” terms for HPO‐based analyses. Our pilot study illustrates how IC thresholds can be used as a proxy for the sensitivity and specificity of HPO gene panels and may improve the analytical bottleneck experienced with classical disease gene panels.

Human Phenotype Ontology gene panels may also reduce reanalysis across multiple disease gene panels. Disease gene panels are often applied to exclude off‐target incidental findings and constrain the analysis broadly to clinically relevant genes. However, many genes are still included which are unrelated to the patient's presentation or conversely fail to include the causative gene in patients with blended phenotypes (Schluter et al., 2022; Schon et al., 2021; Snoeijen‐Schouwenaars et al., 2019). Indeed, while the current study focused on comparing clinical epilepsy panels, 21/38 patients had both IEM and epilepsy panels analysed. This subset of patients had an even greater median number of variants to analyse, and higher rank ratio of the causative variant, than the epilepsy panel alone. The challenge of neatly allocating an individual to a single disease‐track is not uncommon, especially in the context of patients with complex phenotypes such as DEEs. In a large cohort of suspected mitochondrial disease, where seizures were a highly recurrent feature, 63% of the solved families received a non‐mitochondrial diagnosis following WGS (Schon et al., 2021). We designed the epilepsy Phenomodels template with this phenotypic complexity in mind, enabling the manual allocation of any HPO term not captured in the template at the case level. The individualised HPO panels in our study streamlined manual analysis into a single panel, yielded fewer variants and better prioritised the causative gene than the clinical epilepsy panels. A WES/WGS study on white matter disease also found that HPO based analyses reduced the need to reanalyse across related disease tracks and identified atypical/ novel genetic aetiologies (Schluter et al., 2022). HPO panels have the additional advantage of providing up‐to‐date gene associations, enabling reanalysis at the convenience of the clinician.

When phenotypic data is collated in a standardised format, computational phenotype analyses may be applied to automate a significant proportion the currently manual analysis of genomic data. The current clinical norm of unstandardised phenotypic documentation restricts the capacity to leverage this information for broader investigation, which is a significant limitation in the era of precision medicine where multicentre collaborations foster gene discovery and the delineation of epilepsy syndromes (Demarest & Brooks‐Kayal, 2018). Specialists' nuanced insights which will remain essential for accurate interpretation, as highlighted in a study by Akawi et al. (2015) using computational phenotype analyses for gene discovery where they noted that HPO phenotype similarity analyses do not yet appear to capture the complete ‘gestalt’ which is appreciated by experienced clinicians.

Phenotype‐based analyses may also aid our understanding of the complex genetic epilepsies which have proven challenging to interrogate (Thomas & Berkovic, 2014). Polygenic contribution is implicated in common familial epilepsies (International League Against Epilepsy Consortium on Complex, 2018; Leu et al., 2019; Parenti et al., 2020) and has been suggested to influence so called monogenic disease (Demarest & Brooks‐Kayal, 2018; Rubinstein et al., 2015). There is also evidence for a two‐hit model in focal epilepsy (Bennett et al., 2022). One study employing HPO semantic similarity analyses with WES data demonstrated statistical support for multi‐locus pathogenic variation in a patient with severe neurodevelopmental disorder, which included epilepsy (Herman et al., 2021). None of these genes alone were sufficient to explain the patient's blended phenotype. Similarly, another group incorporated HPO‐based gene associations into their WES/WGS prioritisation method in complex blended genetic white matter patients, of which 30% had epilepsy as the main clinical feature, to identify causative variants in more than 1 gene in 6 cases (Schluter et al., 2022).

The current investigation is limited by the retrospective design where only phenotypic information available from the medical records was reviewed, and relatively low case numbers. Individuals included had diagnoses in established epilepsy genes thus the causative variants may be detected at greater sensitivity than causative variants in less‐well established genes. However, our cohort includes DEEs reviewed clinically, reflecting the disease specific care patients typically receive, unlike most other published HPO‐based analytical methods intended to optimise clinical genomic analyses which include patients with diverse diseases and phenotypes.

In conclusion, we have highlighted the potential for phenotype‐based analyses to empower genetic analysis in epilepsy for both research and clinical healthcare purposes. Our pilot study in a clinical workflow introduces a method to efficiently incorporate standardised phenotypic information in the analysis of WES/WGS data which reduced the number of off‐target variants for analysis. The results of the current study have encouraged us to pursue a prospective study to further explore the utility of phenotype‐based analyses in unsolved patients.

## AUTHOR CONTRIBUTIONS

OH conceptualised the study, collected and analysed patient data and drafted the manuscript for intellectual content. TS conceptualised the study, reviewed patient data and revised the manuscript for intellectual content. SB collected patient data and revised the manuscript for intellectual content. CR provided bioinformatics support and revised the manuscript for intellectual content. HS provided bioinformatics support and revised the manuscrip for intellectual content. AW conceptualised the study and revised the manuscript for intellectual content.

## CONFLICT OF INTEREST STATEMENT

The authors declare that they have no competing interests.

## Supporting information


Figure S1.

Figure S2.

Figure S3.

Figure S4.

Table S1.

Table S2.

Table S3.

Figure S1‐S4.

## Data Availability

The data that support the findings of this study are available from the corresponding author upon reasonable request.
