# Supplementary figures and images for: Individualised human phenotype ontology gene panels improve clinical whole exome and genome sequencing analytical efficacy in a cohort of developmental and epileptic encephalopathies
Source: Mol Genet Genomic Med. 2023 Mar 26;11(7):e2167. doi: 10.1002/mgg3.2167 (PMC10337286; doi:10.1002/mgg3.2167)

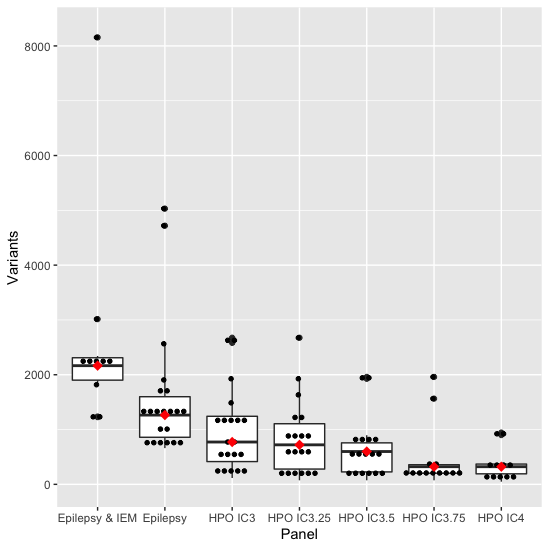

Supplement: Supplementary file 1 — Figure S1. [file MGG3-11-e2167-s002.tiff]

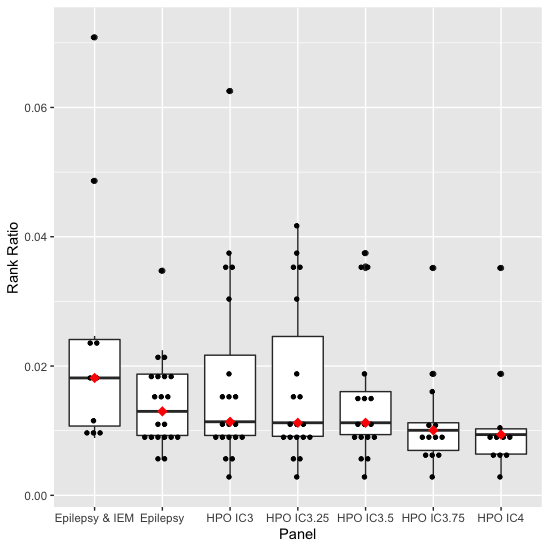

Supplement: Supplementary file 2 — Figure S2. [file MGG3-11-e2167-s006.tiff]

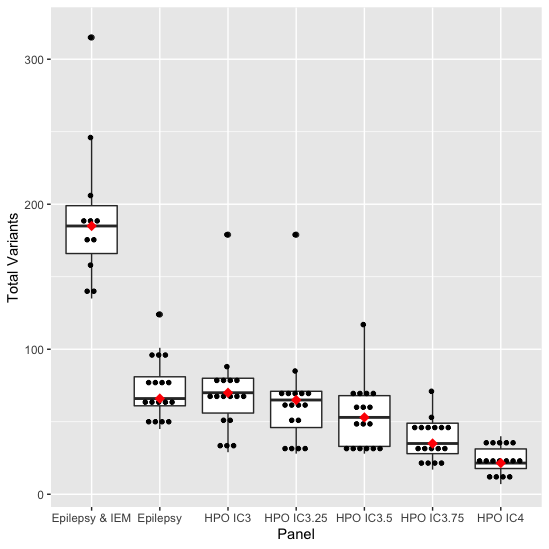

Supplement: Supplementary file 3 — Figure S3. [file MGG3-11-e2167-s003.tiff]

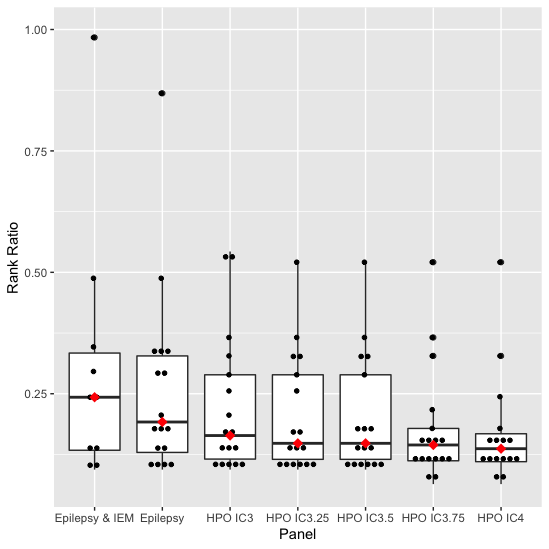

Supplement: Supplementary file 4 — Figure S4. [file MGG3-11-e2167-s008.tiff]
