# Supplementary material for: Individualised human phenotype ontology gene panels improve clinical whole exome and genome sequencing analytical efficacy in a cohort of developmental and epileptic encephalopathies
Source: Mol Genet Genomic Med. 2023 Mar 26;11(7):e2167. doi: 10.1002/mgg3.2167 (PMC10337286; doi:10.1002/mgg3.2167)
Supplement: Supplementary file 8 — Figure S1‐S4. [file MGG3-11-e2167-s005.docx]

**Supplementary figure 1.** Total WGS variants in the clinical Epilepsy and IEM gene panels, compared with the individualised HPO gene panels. Red dots indicate median values. The box contains the interquartile range. Values outside the whiskers are outliers.

**Supplementary figure 2.** WGS rank ratio in the clinical Epilepsy and IEM gene panels, compared with the individualised HPO gene panels. Red dots indicate median values. The box contains the interquartile range. Values outside the whiskers are outliers.

**Supplementary figure 3.** Total WES variants in the clinical Epilepsy and IEM gene panels, compared with the individualised HPO gene panels. Red dots indicate median values. The box contains the interquartile range. Values outside the whiskers are outliers.

**Supplementary figure 4.** WES rank ratio in the clinical Epilepsy and IEM gene panels, compared with the individualised HPO gene panels. Red dots indicate median values. Extreme outlier 1.737 from the Epilepsy and IEM gene panel has been removed for visualization purposes. The box contains the interquartile range. Values outside the whiskers are outliers.

**Supplementary table 1.** Motivation for terms included in the epilepsy phenotyping template.

**Supplementary table 2**. WGS cohort causative genes and individualised IC **≥** 3 panel data. IC3_GeneNo: number of genes included in gene panel. IC3_variants: number of variants included in the gene panel. IC3_rank: rank of the causative variant in the gene panel. IC3_rank_ratio: rank ratio of the causative variant in the gene panel. IC3_NoHPO: number of HPO terms included in the gene panel.

**Supplementary table 3**. WES cohort causative genes and individualised IC **≥** 3.75 panel data. IC3.75_GeneNo: number of genes included in gene panel. IC3.75_variants: number of variants included in the gene panel. IC3.75_rank: rank of the causative variant in the gene panel. IC3.75_rank_ratio: rank ratio of the causative variant in the gene panel. IC3.75_NoHPO: number of HPO terms included in the gene panel.
